# Nonattendance at Scheduled Appointments in Outpatient Clinics Due to COVID-19 and Related Factors in Taiwan: A Health Belief Model Approach

**DOI:** 10.3390/ijerph18094445

**Published:** 2021-04-22

**Authors:** Yi-Ping Hsieh, Cheng-Fang Yen, Chia-Fen Wu, Peng-Wei Wang

**Affiliations:** 1 Department of Social Work, College of Nursing and Professional Disciplines, University of North Dakota, Grand Forks, ND 58202, USA; yiping66@gmail.com; 2 Department of Psychiatry, School of Medicine, College of Medicine, Kaohsiung Medical University, Kaohsiung 807, Taiwan; p03132006@gmail.com (C.-F.Y.); pino3015@hotmail.com (C.-F.W.); 3 Department of Psychiatry, Kaohsiung Medical University Hospital, Kaohsiung 807, Taiwan

**Keywords:** COVID-19 pandemic, health belief, self-efficacy, medical appointment

## Abstract

During the COVID-19 pandemic, the number of hospital visits and attendance at scheduled appointments have dropped significantly. We used the health belief model (in three dimensions) to examine the determinants of non-attendance of scheduled appointments in outpatient clinics due to the COVID-19 pandemic. Participants in Taiwan (*n* = 1954) completed an online survey from 10 April 10 to 23 April 2020, which assessed how people perceived and responded to the outbreak of a fast-spreading infectious disease. We performed both univariate and multivariate logistic regression to examine the roles of cognitive, affective, and behavioral health belief constructs in nonattendance at scheduled appointments. The results indicated that individuals who perceived high confidence in coping with COVID-19 were less likely to miss or cancel their doctor’s appointments, whereas individuals who reported high anxiety and practiced more preventive health behaviors, including avoiding crowded places, washing hands more often, and wearing a mask more often, were more likely to miss or cancel their appointments due to the COVID-19 pandemic. Non-heterosexual participants had a lower rate of nonattendance at scheduled appointments compared with heterosexual ones. The study results increase our understanding of the patients’ cognitive health beliefs, psychological distress, and health behaviors when assessing adherence to medical appointments during a pandemic.

## 1. Introduction

### 1.1. Coronavirus Disease 2019 Pandemic

Coronavirus disease 2019 (COVID-19) is a novel viral disease that has become a global public health concern. The country Centers for Disease Control and Prevention (CDC) [1] has identified how the virus spreads and has recommended strategies for prevention. COVID-19 is thought to mainly spread from person-to-person, most commonly through close contact (within 6 feet). The virus travels through respiratory droplets released in the air when an infected person coughs, sneezes, or talks. No vaccine was available to prevent COVID-19, and it has spread rapidly worldwide, with a 2–5% mortality rate [1,2]. Asymptomatic individuals may spread COVID-19; older adults and people with underlying conditions seem to be at a higher risk of developing serious complications [1,3,4]. Since 31 December 2019, and as of 17 October 2020, 39,400,032 confirmed cases, including 1,105,353 deaths, have been reported worldwide [5]. Various strategies for COVID-19 management have been implemented globally, which focus on minimizing the transmission and spread of infection and providing supportive care for infected individuals. To prevent COVID-19 importation and outbreaks, the Taiwanese government took rapid action, including border control, case identification using new data and technology, quarantining suspicious cases, allocating resources, and reassuring and educating the public [6]. After experiencing the severe acute respiratory syndrome (SARS) outbreak in 2002, the government and general public of Taiwan could quickly respond to the COVID-19 pandemic and took precautionary measures seriously. In Taiwan, the CDC and Central Epidemic Command Center announced policies and regulations and educated the public about COVID-19 through various media sources. The Taiwanese public has adhered well to the control measures, including mask-wearing, maintaining hand and respiratory hygiene, temperature monitoring, avoiding public gatherings, and maintaining social distancing and the quarantine of individuals who are infected or at high-risk [7].

### 1.2. Trends in Outpatient Visits

To avoid gatherings, many people reduced or stopped normal activities, including travelling, going to restaurants and stores and visiting outpatient clinics or hospitals. Due to the concerns of the COVID-19 pandemic, the overall number of hospital visits in Taiwan decreased by 14% in the first quarter of 2020 [8]. In the United States, the number of outpatient visits decreased by approximately 60% from early in the pandemic to early April 2020 [9]. In the United Kingdom, similar moderating trends in emergency department visits were observed nationwide, with a decline of 25% within 7 days of the national lockdown [10], and general practitioner appointments decreased by 30% in March 2020 from the previous year [11]. The potential reason for the decrease in outpatient visits is to avoid hospital visits due to the fear of contracting COVID-19 and the increased use of pharmacological reperfusion due to COVID-19 [12]. The fear of COVID-19 compelled people with life-threatening emergencies to stay at home [12], and patients were willing to self-treat and monitor their health at home because they believe that emergency departments (EDs) were repurposed for COVID-19, and that they were more likely to be exposed to COVID-19. After containment measures against COVID-19 were implemented, a substantial decrease in the number of admissions to intensive coronary care units was observed in France [13]. These patients indicated two main reasons for missing appointments: the fear of being infected (38%) and the fear of disturbing their doctor during the pandemic (28%). The fear of potential in-hospital infection and the long wait before consultation in an overcrowded emergency room were suggested as reasons for the decreased admission rate [13]. Other factors including financial risk, which increased due to the sudden rise in unemployment and the loss of health insurance, may cause patients to opt out of ED visits [14].

COVID-19 affects not only nonattendance at scheduled appointments in outpatient clinics, but also other aspects. For example, the total number of outpatients, hospital admissions, and surgeries conducted during the pandemic (March and April 2020) were also significantly smaller than those in the corresponding months of the previous year on orthopedic surgery practice [15]. The impacts included a 20–29% reduction in outpatients, 22–37% reduction in admissions, and 18–35% reduction in surgeries. Similarly, COVID-19 has also changed the daily practice of dermatologists dramatically with a significant decrease in the number of visits in 2020, compared to 2018 and 2019 [16]. Those with non-emergent dermatosis were unlikely to visit the dermatology clinic in Taiwan during the pandemic, in contrast to patients with skin cancer. Moreover, COVID-19 has had great impacts on all forms of mental health care—inpatient, community, residential and outpatient across seven countries including Taiwan [17], especially for services which depended on face-to-face and hospital-based care. There was a general decrease in demand for mental health care during the initial and acceleration phase of the pandemic. Rehabilitation and social services for mental illness have also been affected or ceased. Routine care for chronic disease, such as diabetes, chronic obstructive pulmonary disease, and hypertension, were the most impacted conditions in the COVID-19 pandemic due to reduction in access to care across countries [18]. In Taiwan, there was a statistically significant decrease in the old elderly patients (aged above 75) with chronic diseases returning to hospital for medicines at early stage of outbreak [19].

### 1.3. Health Belief Model and Preventive Health Behavior

Understanding the public’s behaviors, beliefs, and perceptions is critically important in assessing adherence to health advice [20] and medical appointments. The health belief model (HBM) is one of the most widely used frameworks in health behavior research [21]. Health-related behaviors in the face of a disease or the risk of contracting a disease are determined by the health beliefs and cues to action of an individual [22]. These primary constructs include perceived susceptibility, perceived severity, self-efficacy, and expectations of the behavior (perceived benefits and barriers) [21,22]. Based on the HBM, we identified three dimensions of these health belief constructs—cognitive construct, affective construct, and behavioral construct of health belief. First, the cognitive constructs of health belief include perceived susceptibility, perceived severity, and self-efficacy (confidence). Perceived susceptibility refers to personal beliefs about the likelihood of contracting a disease [21]. Individuals must believe that there is a high possibility of contracting COVID-19 before they consider taking precautions. Perceived severity refers to personal beliefs about the seriousness of clinical and social consequences of being infected [21]. For example, an individual must believe that there is a high possibility of severe short-term (death) or long-term (organ damage) consequences after contracting COVID-19 before taking the disease seriously. Moreover, media reports may produce fear, worry, and anxiety in the public. Self-efficacy refers to the confidence in one’s ability to act according to social cognitive theory [23], and it is a part of the HBM [21,24]. Individuals educated about COVID-19 may have high self-efficacy and confidence in coping with COVID-19. Second, the affective constructs of health belief include worry and anxiety about the diseases. Third, the behavioral constructs of health belief involve both perceived benefits and barriers. Perceived benefits refer to personal beliefs about the efficacy of actions to reduce the disease threat [21]. For example, individuals may not practice the recommended precautions, including washing hands more often, maintaining social distancing, and wearing facemasks or coverings, unless the perceived benefits of these actions reduced the threat or risk to health. Perceived barriers refer to personal beliefs about the negative externalities or costs of the preventive health actions while weighing the expected benefits [21]. For example, although wearing facemasks can reduce the risk of contracting COVID-19, it can be unpleasant, inconvenient, and expensive or has side effects, including difficulty in breathing and impairment of nonverbal communication, which encourages noncompliance to regulations.

### 1.4. Nonattendance in Outpatient Clinics among Sexual Minority Individuals

COVID-19 may have a disproportionate impact on physical and mental health of sexual minorities [25]. Sexual minority individuals already have a higher burden of chronic health problems such as human immunodeficiency virus (HIV) infection [26], cancer [27], diabetes [28] and hypertension [29] than heterosexuals before the COVID-19 pandemic. As demonstrated by the minority stress model [30], negative attitude toward sexual minority in the society may worsen the health of sexual minority individuals. Sexual minority individuals are less likely to seek health care due to stigma and discrimination [31]. Furthermore, compared with heterosexual individuals, the COVID-19 pandemic disproportionately impacted mental health [32] and economic capability [33] of sexual minority individuals and thus may compromise their ability to access health care services. A recent study reported that the COVID-19 pandemic resulted in interruptions of HIV treatment in sexual minority individuals [34]. Whether there is difference in nonattendance at scheduled appointments in outpatient clinics during the COVID-19 pandemic between sexual minority and heterosexual individuals’ warrants study.

### 1.5. Aims of This Study

This study used HBM to examine the determinants of nonattendance at scheduled appointments in outpatient clinics due to the COVID-19 pandemic, which describe healthcare decision-making behavior in this uncertain time. The primary concepts of health beliefs were considered in terms of three dimensions: cognitive, affective, and behavioral constructs. The cognitive constructs of health belief include perceived susceptibility to COVID-19, perceived severity of COVID-19, having enough knowledge and information on COVID-19, and confidence in coping with COVID-19. The affective constructs of health belief include worry about COVID-19 and general anxiety. The behavioral constructs of health belief include avoiding crowded places, washing hands more often, and wearing a mask more often. Regarding the cognitive constructs of health belief, we hypothesized that people who perceived higher levels of susceptibility and severity to COVID-19 were more likely to miss scheduled appointments in clinics due to the COVID-19 pandemic, whereas people with enough knowledge and information on COVID-19 and confidence in coping with it were less likely to miss appointed appointments due to the pandemic. Regarding the affective constructs of health belief, we hypothesized that people who were worried about COVID-19 and had higher levels of anxiety were more likely to miss scheduled appointments in clinics. Regarding the behavioral constructs of health belief, we hypothesized that people who avoided crowed places, washed hands more often, and wore masks more often during the pandemic were more likely to miss scheduled appointments at the clinic. We also compared the rates of nonattendance at scheduled appointments between heterosexual and non-heterosexual individuals and the moderating effect of sexual orientation in the association between HBM constructs and nonattendance.

## 2. Methods

### 2.1. Participants and Procedures

We recruited 1954 participants through a Facebook advertisement from 10 April to 23 April 2020. Facebook users who were ≥20 years old and lived in Taiwan were eligible for this study. Each participant completed an online survey, which assessed how the general public perceived and responded to the outbreak of a fast-spreading infectious disease [35].

Our Facebook advertisement was posted on Facebook as “news feeds,” which is a constantly updating list of stories and information highlights. Compared with other Facebook advertising locations, such as the right column, we targeted users’ news feed advertisements because it is the most effective way to recruit research participants [36]. We targeted Facebook users by location (Taiwan) and language spoken (Chinese). Facebook’s advertising algorithm determined our audience based on our criteria. The Facebook advertisement included a headline, main text, pop-up banner, and link to the research questionnaire website. To ensure diversity of sexual orientation among participants during the recruitment, we posted the advertisements on three Facebook pages of Taiwanese health promotion and counseling centers for lesbian, gay, and bisexual individuals. Facebook has a younger and more progressive audience than the general population [37]. For example, in our sample only 1.4% of adults were elderly (65 years old or above), which is much less than the percentage in the general population in Taiwan (16%) [38]. While 89% of the participants in our sample have a high education level (college or above), the corresponding percentage in the general population in Taiwan was 47% [38]. While 67% of the participants in our sample were female, the percentage in the general population is 50% [38].

This study was approved by the Institutional Review Board (IRB) of Kaohsiung Medical University Hospital (KMUHIRB-EXEMPT(I) 20200011). IRB indicated that this study did not require written informed consent because participation was voluntary and survey responses were anonymous. No incentive was given to the participants. For participants to learn more about COVID-19, we provided useful links from the Taiwan CDC, Kaohsiung Medical University Hospital, and Medical College of National Cheng Kung University.

### 2.2. Measures

The questionnaire survey was conducted in Mandarin. The measures were listed below.

#### 2.2.1. Nonattendance at Scheduled Appointments in Outpatient Clinics

We used the following question to assess participant nonattendance at scheduled appointments in outpatient clinics: “In the past 7 days, have you ever missed a doctor appointment?” The responses were categorized as 0, “no” or “yes, but not due to COVID-19” and 1, “yes, due to COVID-19.”

#### 2.2.2. HBM Constructs

We used the questionnaire developed by Liao et al. [39] to measure the cognitive, affective and behavioral HBM constructs in the context of the COVID-19 pandemic. The original questionnaire was in Mandarin and developed for measuring cognitive, affective and behavioral constructs of the HBM among people in Hong Kong during the 2009 influenza A/H1N1 pandemic [39]. Given that there are similar characteristics of respiratory infectious diseases between COVID-19 and influenza A/H1N1 and that the questionnaire of Liao et al. [39] contained the HBM constructs we aimed to measure during the COVID-19 pandemic, the questionnaire was suitable for this study. We also used the same cutoffs recommended by the original study to dichotomize the variables.

We measured four cognitive constructs of the HBM. First, we assessed the perceived susceptibility to COVID-19 using the question “What do you think are your chances of contracting COVID-19 over the next 1 month compared with others outside your family?” [39]. This question was rated from 1 (no chance of contracting COVID-19) to 7 (guaranteed to contract COVID-19). Scores of 5 or more indicated high perceived susceptibilities and less than 5 indicated low perceived susceptibilities [39]. Second, we assessed the perceived severity of COVID-19 relative to SARS using the question “How serious is COVID-19 relative to SARS?” [39]. This question was rated from 1 (COVID-19 is much less serious) to 5 (COVID-19 is much more serious). Scores of 4 or more indicated high perceived severity and less than 4 indicated low perceived severity [39].

Third, a barrier in the HBM includes a lack of necessary knowledge [40]. We assessed the level of necessary knowledge using the yes/no question “Do you think you have sufficient knowledge and information on COVID-19?” Fourth, we assessed the perceived self-confidence in coping with COVID-19 using the question “How confident are you that you can cope well with COVID-19?” [41]. This question was rated from 1 (not confident at all) to 5 (very confident). Scores of 3 or more indicated high self-confidence levels and less than 3 indicated low self-confidence levels.

We measured two affective constructs in the context of the COVID-19 pandemic. First, we assessed the level of worry about COVID-19 through the question “Please rate how worried you are currently toward COVID-19” [39]. The response was rated from 1 (very mild) to 10 (very severe). Scores of 6 or more indicated high levels of worry, and scores less than 6 indicated low levels of worry. Second, we measured general anxiety in the past 1 week using a validated state anxiety scale from the State-Trait Anxiety Inventory [42]. Participants were asked to rate their feelings in response to 10 statements, including feelings of being rested, contented, comfortable, relaxed, pleasant, anxious, nervous, jittery, highly strung, and over-excited and “rattled” on a 4-point Likert scale (1 = not at all, 2 = sometimes, 3 = moderately so, 4 = very much so) [39,42,43]. Several items were reverse coded. Higher scores indicated greater anxiety. Cronbach’s α for these items was 0.92. Scores of 23 (the median) or more indicated high levels of anxiety, and scores less than 23 indicated low levels of anxiety.

The behavioral constructs assessed focused on whether the participants practiced preventive measures daily. Participants were asked if they (1) avoided crowded places, (2) washed their hands more often, and (3) wore a mask more often in the past 7 days [39]. The responses were categorized as 0, “no” or “yes, but not due to COVID-19” or 1, “yes, due to COVID-19.” Participants who responded “yes, due to COVID-19” were classified as practicing preventive measures daily [39].

#### 2.2.3. Demographic Characteristics

We collected demographic data, including gender, age, sexual orientation (heterosexual vs. non-heterosexual), and education level (university qualifications or above vs. high school qualifications or below).

### 2.3. Statistical Analysis

Data analysis was performed using SPSS 22.0 statistical software (SPSS Inc., Chicago, IL, USA). Demographic characteristics; cognitive, affective, and behavioral constructs of health beliefs related to COVID-19; and general anxiety were compared between respondents who did or did not experience nonattendance at scheduled appointments in outpatient clinics due to COVID-19 by using univariate logistic regression with the crude odds ratio (cOR). All potential predictive variables identified from the first step were included in the multivariate logistic regression models with the adjusted odds ratio (aOR) calculated to determine independent predictors of nonattendance at scheduled appointments. According to Senaviratna and Cooray [44], we used Tolerance, Variance Inflation Factor (VIF) and Condition Index to examine the multicollinearity of logistic regression analysis. The values of Tolerance 0.1 or less, VIF above 2.5 and Condition Index above 30 indicate a concern for multicollinearity among variables. The moderating effect of sexual orientation on the association between HBM constructs and nonattendance was also examined. The odds ratio (OR), *p* value, and 95% confidence interval (CI) were used to indicate significance. A two-tailed *p* value of < 0.05 indicated statistical significance.

## 3. Results

### 3.1. Summaries of Descriptive Analysis

Data from 1954 patient respondents (women: 66.8%, men: 33.2%; mean age = 37.87 years) were analyzed; 77 of the original 2031 participants were excluded due to missing data. In total, 201 (10.3%) respondents reported nonattendance at scheduled appointments in outpatient clinics in the past week. Table 1 shows the results of univariate logistic regression analysis of the association between demographic characteristics with nonattendance at scheduled appointments in outpatient clinics. Older age was significantly associated with nonattendance at scheduled appointments. Heterosexual respondents were more likely to have nonattendance at scheduled appointments than nonheterosexual respondents. No difference was found in nonattendance between genders or between respondents with high and low education levels.

### 3.2. Comparisons Constructs of Health Beliefs between Attendance and Nonattendance at Scheduled Appointments

Table 2 presents the results of univariate logistic regression analysis of the associations of cognitive, affective, and behavioral constructs of health beliefs related to the COVID-19 pandemic with nonattendance at scheduled appointments in outpatient clinics. The results indicated that respondents who perceived high susceptibility to COVID-19 and had low confidence in coping with COVID-19 were more likely to have nonattendance at scheduled appointments than those who perceived low susceptibility to COVID-19 and who had high confidence in coping with COVID-19. Respondents who were highly worried about COVID-19 and had high general anxiety were more likely to have nonattendance than those who had low levels of worry and low general anxiety. Respondents who avoided crowded places, washed hands more often, or wore a mask more often were more likely to have nonattendance at scheduled appointments than those who did not practice these protective behaviors. However, perceived COVID-19 severity relative to SARS and knowledge on COVID-19 were not significantly associated with nonattendance at scheduled appointments.

### 3.3. Factors Associated with Nonattendance at Scheduled Appointments

Variables that were significantly associated with nonattendance at scheduled appointments in the univariate logistic regression model were included in the multivariate logistic regression model (Table 3). The multicollinearity among variables have not be identified because all Tolerances were larger than 0.1, VIFs were less than 2.5 and Condition Indexes were less than 30 (Supplementary Table S1). The results indicated that older age, heterosexuality, low confidence in coping with COVID-19, high general anxiety, avoiding crowded places, washing hands more often, and wearing a mask more often were significantly associated with nonattendance at scheduled appointments. However, perceived susceptibility to and worry about COVID-19 became not significantly associated with nonattendance at scheduled appointments.

### 3.4. Moderating Effect of Sexual Orientation on the Assocaition between HBM Construcs and Nonattendance

The interactions between sexual orientation and HBM constructs that were significantly associated with nonattendance were put into logistic regression model to examine the moderating effect of sexual orientation (Table 4). The results demonstrated that the interaction variables were not significantly associated with nonattendance, indicating no moderating effect of sexual orientation.

## 4. Discussion

### 4.1. Principle Results

This study contributes to the understanding of how people make healthcare decisions (keeping appointments) based on what they believed in, how they felt and reacted to the pandemic, and the preventive health behaviors they practiced. Uniquely, because Taiwan responded to the pandemic very early in January 2020 with border control, case identification using technology, quarantine of suspicious cases, resource allocation, etc., there was no community spread and no lockdown or stay-home order. Therefore, the nonattendances of scheduled appointments were not due to lockdown but because of individuals’ health beliefs. A total of 10.3% of the participants (heterosexual: 12.1%, non-heterosexual: 5.4%) in our study reported that they missed scheduled appointments in the past week because of the COVID-19 pandemic, suggesting that patients preferred to self-treat and monitor their health at home due to the anxiety or fear of exposure to COVID-19 at the clinic or hospital. However, the failure to keep a scheduled appointment may jeopardize patient health and well-being [45,46]. Understanding patients’ beliefs, psychological distress, and health behaviors is therefore important in assessing adherence to medical appointments.

This study is the first to investigate the determinants of nonattendance at scheduled appointments in outpatient clinics due to the COVID-19 pandemic using HBM in Taiwan. Only one study has investigated appointment-keeping behavior for a potentially fatal chronic disease using HBM [47]. Our findings indicate that all three constructs of health belief (cognitive, affective, and behavioral) were associated with nonattendance at scheduled appointments due to the COVID-19 pandemic. After considering age, sexual orientation, and other health belief constructs, individuals who perceived high confidence in coping with COVID-19 were less likely to miss or cancel their doctor’s appointments, which supported our hypothesis. If individuals perceive high efficacy of prevention measures, they are more likely to adhere to these prevention measures [48]. If the government and community can emphasize and increase people’s perceived efficacy of coping with COVID-19, it will reduce the threat and fear of the disease [23] and ensure appointment-keeping and adhere to health advice.

### 4.2. Three HBM Constructs and Nonattendance at Schedule Appointments

Regarding the cognitive constructs of health belief, individuals who had high confidence in coping with COVID-19 were less likely to miss or cancel doctor’s appointments. The confidence of the coping may be considered not only in the individual level but also the contextual levels. In very early stage of the pandemic in 20 January 2020, the Central Epidemic Command Center (CECC) in Taiwan was activated and rapidly responded to the pandemics. With the efforts of the CECC and all residents in Taiwan (total population is 23.8 million in 2020), there was no community spread and the number of confirmed cases until 23 April 2020 (the end of our survey time) was quite low (confirmed cases = 427, deaths = 6), and most of the cases were imported cases [49]. Moreover, CECC held the press conference every day to provide the most accurate, transparent and updated information and education to the public and answer any questions from the reports. Therefore, the majority of general public viewed CECC as trustworthy and believed the health system in Taiwan has the capacity in handling the pandemic situations and treatments without great concerns of collapses. It might contribute to the confidence in coping with COVID-19.

Regarding the affective constructs of health belief, our results indicated that individuals who reported high anxiety were more likely to miss or cancel their appointments due to the COVID-19 pandemic. Given the continuously increasing number of infected cases, rapid spread of the outbreak globally, and uncertainty, people are likely to experience psychological stress and mental health challenges. The spread of rumors on social media about disease severity may induce greater fear, worry, stigma, and discrimination against victims of infectious disease and increase anxiety levels [50,51]. In this study, approximately 63% of the respondents reported high levels of worry about COVID-19, and 48% respondents reported high levels of general anxiety. Furthermore, high levels of worry were associated with nonattendance at appointments; however, the relationship became insignificant after considering other constructs of health belief. By contrast, general anxiety was the strongest affective construct that explained patients’ missing appointments after considering other confounding factors. When individuals felt highly anxious, they tended to stay in the comfort zone without taking the risks of attending doctor’s appointments, especially during the pandemic which was full of uncertainty and increased anxiety.

Regarding the behavioral constructs of health belief, individuals who practiced more preventive health behaviors, such as avoiding crowded places, washing hands more often, and wearing a mask more often, were more likely to miss or cancel their appointments due to the COVID-19 pandemic, after considering other constructs of health belief. Because there was no vaccine for COVID-19 in 2020, the use of preventive measures, including social distancing, maintaining hand hygiene, and wearing a facemask, has become an alternate strategy for managing the spread of the pandemic [52]. Patients who are not highly concerned about the pandemic may become highly concerned because of the numerous recommended preventive strategies, including washing hands for 20 s (the length of singing a birthday song twice) many times a day or using sanitizers often, requirement of wearing a facemask, wiping surfaces very often, maintaining social distancing, avoiding shaking hands or sharing objects with others, entering and exiting a building or room using different doors, and working from home. These preventive strategies might be associated with reinforced beliefs about the susceptibility to and severity of the disease, including swine flu [53] and COVID-19. Our results support the hypothesis that the more the preventive strategies were followed by the patients, the more concerns they had of the COVID-19 pandemic, and the more they were likely to miss or cancel the scheduled appointments.

### 4.3. Role of Sexual Orientaiton in Nonattendance

The present study demonstrated that non-sexual individuals had a lower rate of nonattendance than heterosexual ones, whereas no moderating effect of sexual orientaiton was found on the association between HBM constructs and nonattendance. As mentioned before, research has revealed that non-heterosexual individuals may have higher rates of chronic health problems [26,27,28,29] that may make regular attendance at the clinics become necessary. The quality national health insurance provides every citizen in Taiwan health care at a reasonable cost. Given that the present study did not examine what departments the scheduled appointments the participants missed were in nor what kinds of physical and psychiatric illnesses the participants had, we could not determine whether the difference in non-attendance between non-heterosexual and heterosexual participants came from the varieties of illnesses and severities.

### 4.4. Limitations

Although this study provides insights into the roles of three health belief constructs to explain non-attendance at scheduled appointments due to the COVID-19 pandemic, there are several limitations. First, because of the cross-sectional design, causal conclusions and inferences between and among variables cannot be drawn. Second, the number of confirmed cases, the emergence and spread of the disease, death rates, and national or local responses may vary globally, and the results of this study from a Taiwanese sample may not be generalizable to other populations, especially because Taiwan has a relatively lower number of confirmed cases and does not show signs of community spread. Third, factors including stigma or discrimination toward people who are infected by COVID-19 may cause individuals to hide their infection and miss the scheduled appointments. Future studies may examine the role of social stigma in the context of the pandemic. Fourth, we did not separate the participants into those who miss or cancelled scheduled appointments, those who attended scheduled appointments, and those who did not have scheduled appointments. The HBM constructs identified to be associated with nonattendance in the present study could only discriminate between the nonattendance group and other two groups. Lastly, because Facebook has a younger and more progressive audience than the general population [37], the results in our study may not be generalizable to the general population, especially the elderly.

## 5. Conclusions

The study results increase our understanding of the patients’ cognitive health beliefs, psychological distress, and health behaviors when assessing adherence to medical appointments during a pandemic. Moreover, when the government or community can provide and deliver accurate and trustworthy information and education to the public and allocate resources, it may increase patients’ self-efficacy and confidence in coping with COVID-19 and decrease their anxiety levels, which in turn will reduce the unreasonable fear of the disease and ensure appointment-keeping and adhere to health advice. In addition, when patients took the pandemic very seriously by strictly following the preventive measures/strategies, they also tended to eliminate any risks of exposure to the COVID-19 by not attending doctor’s appointments unless it is an emergency. Alternative options should be developed and provided to patients to ensure their health and well-being are not jeopardized during the pandemic.

## Figures and Tables

**Table 1:** Comparisons of Demographic Characteristics between Respondents with and Without Nonattendance at Scheduled Appointments: Univariate Logistic Regression Model (*n* = 1954).

Variables	Nonattendance		Univariate Logistic Model			
	No	Yes	B	cOR	95% CI	*p* Value
Gender, *n* (%)						
Female (*n* = 1305)	1161 (89.0)	144 (11.0)	−0.253	0.776	0.562–1.071	0.124
Male (*n* = 649)	592 (91.2)	57 (8.8)				
Age (years), mean (SD)	37.5 (10.8)	40.8 (10.4)	0.027	1.027	1.014–1.041	<0.001
Sexual orientation						
Nonheterosexual (*n* = 533)	504 (94.6)	29 (5.4)	0.873	2.393	1.593–3.595	<0.001
Heterosexual (*n* = 1421)	1249 (87.9)	172 (12.1)				
Education level, *n* (%)						
University or above (*n* = 1736)	1561 (89.9)	175 (10.1)	0.189	1.208	0.779–1.872	0.398
High school or below (*n* = 218)	192 (88.1)	26 (11.9)				

CI: Confidence interval; cOR: Crude odds ratio; SD: Standard deviation.

**Table 2:** Comparisons of Health Beliefs and General Anxiety between Respondents with and Without Nonattendance at Scheduled Appointments: Univariate Logistic Regression Model (*n* = 1954).

Factors	Nonattendance		Univariate Logistic Model			
	No	Yes	B	cOR	95% CI	*p*
Cognitive constructs of health belief						
Perceived susceptibility to COVID-19, *n* (%)						
Low (*n* = 1608)	1459 (90.7)	149 (9.3)	0.549	1.732	1.233–2.432	0.002
High (*n* = 346)	294 (85.0)	52 (15.0)				
Perceived COVID-19 severity relative to SARS, *n* (%)						
Low (*n* = 575)	522 (90.8)	53 (9.2)	0.169	1.184	0.851–1.647	0.316
High (*n* = 1379)	1231 (89.3)	148 (10.7)				
Having enough knowledge and information about COVID-19, *n* (%)						
No (*n* = 191)	174 (91.1)	17 (8.9)	0.176	1.193	0.708–2.008	0.507
Yes (*n* = 1763)	1579 (89.6)	184 (10.4)				
Confidence in coping with COVID-19, *n* (%)						
Low (*n* = 268)	221 (82.5)	47 (17.5)	−0.749	0.473	0.331–0.675	<0.001
High (*n* = 1686)	1532 (90.9)	154 (9.1)				
Affective construct of health belief						
Worry about COVID-19, *n* (%)						
Low (*n* = 726)	671 (92.4)	55 (7.6)	0.498	1.646	1.190–2.278	0.003
High (*n* = 1228)	1082 (88.1)	146 (11.9)				
General anxiety, *n* (%)						
Low (*n* = 1011)	940 (93.0)	71 (7.0)	0.750	2.117	1.562–2.869	<0.001
High (*n* = 943)	813 (86.2)	130 (13.8)				
Behavioral constructs of health belief						
Avoiding crowded places, *n* (%)						
No (*n* = 367)	355 (96.7)	12 (3.3)	1.386	3.999	2.206–7.251	<0.001
Yes (*n* = 1587)	1398 (88.1)	189 (11.9)				
Washing hands more often, *n* (%)						
No (*n* = 443)	432 (97.5)	11 (2.5)	1.731	5.649	3.046–10.474	<0.001
Yes (*n* = 1511)	1321 (87.4)	190 (12.6)				
Wearing a mask more often, *n* (%)						
No (*n* = 443)	429 (96.8)	14 (3.2)	1.465	4.328	2.487–7.531	<0.001
Yes (*n* = 1511)	1324 (87.6)	187 (12.4)				
Yes (*n* = 1587)	1398 (88.1)	189 (11.9)				

CI: Confidence interval; COVID-19: Coronavirus disease 2019; cOR: Crude odds ratio; SARS: Severe acute respiratory syndrome.

**Table 3:** Factors Related to Nonattendance at Scheduled Appointments: Multivariate Logistic Regression Model.

Factors	B	aOR	95% CI	*p*
Age	0.021	1.021	1.007–1.036	0.004
Heterosexuals ^a^	0.572	1.772	1.153–2.724	0.009
Cognitive constructs of health belief				
Perceived high susceptibility to COVID-19 ^b^	0.273	1.315	0.914–1.890	0.140
High confidence in coping with COVID-19 ^c^	−0.393	0.675	0.459–0.993	0.046
Affective construct of health belief				
High worry about COVID-19 ^d^	0.057	1.059	0.741–1.514	0.753
High general anxiety ^e^	0.517	1.677	1.207–2.330	0.002
Behavioral constructs of health belief				
Avoiding crowded places	0.632	1.882	1.005–3.524	0.048
Washing hands more often	1.085	2.960	1.531–5.725	0.001
Wearing a mask more often	0.777	2.174	1.199–3.940	0.011

^a^: Nonheterosexual as the reference; ^b^: Low susceptibility to COVID-19 as the reference; ^c^: Low confidence in coping with COVID-19 as the reference; ^d^: Low worry about COVID-19 as the reference; ^e^: Low general anxiety as the reference. aOR: Adjusted odds ratio; CI: Confidence interval; COVID-19: Coronavirus disease 2019.

**Table 4:** Moderating Effect of Sexual Orientation on the Assocaition between HBM Construcs and Nonattendance ^a^.

Variables	B	aOR	95% CI	*p*
Heterosexuals × Confidence in coping with COVID-19	0.187	1.206	0.393–3.705	0.744
Heterosexuals × Gneral anxiety	−0.192	0.825	0.344–1.981	0.668
Heterosexuals × Avoiding crowded places	0.041	1.042	0.245–4.440	0.955
Heterosexuals × Washing hands	−0.231	0.794	0.146–4.319	0.789
Heterosexuals × Wearing a mask	0.429	1.536	0.356–6.628	0.565

^a^: Controlling for age, sexual orientaiton, susceptibility to COVID-19, confidence in coping with COVID-19, worry about COVID-19, gneral anxiety, worry about COVID-19, avoiding crowded places, washing hands, and wearing a mask. aOR: Adjusted odds ratio; CI: Confidence interval; COVID-19: Coronavirus disease 2019.

## Data Availability

The data will be available upon reasonable request to the corresponding authors.

## Supplementary Materials

The following are available online at https://www.mdpi.com/article/10.3390/ijerph18094445/s1, Table S1: The result for multicollinearity among variables in Multivariate Logistic Regression Model
