# Supplementary material for: Nonattendance at Scheduled Appointments in Outpatient Clinics Due to COVID-19 and Related Factors in Taiwan: A Health Belief Model Approach
Source: Int J Environ Res Public Health. 2021 Apr 22;18(9):4445. doi: 10.3390/ijerph18094445 (PMC8122706; doi:10.3390/ijerph18094445)
Supplement: Supplementary file 1 [file ijerph-18-04445-s001.zip › ijerph-1120990-supplementary.pdf]

Supplementary table 1 The result for multicollinearity among variables in Multivariate Logistic Regression Model

	Condition Index	Tolerance	VIF
Age	2.658	0.894	1.119
Heterosexuals[a]	3.052	0.894	1.119
Cognitive constructs of health belief			
Perceived high susceptibility to COVID-19[b]	3.202	0.961	1.040
High confidence in coping with COVID-19[c]	3.869	0.918	1.089
Affective construct of health belief			
High worry about COVID-19[d]	5.229	0.885	1.130
High general anxiety[e]	7.042	0.902	1.109
Behavioral constructs of health belief			
Avoiding crowded places	7.485	0.814	1.229
Washing hands more often	8.537	0.725	1.379
Wearing a mask more often	16.741	0.721	1.387
